# Continuous Spatial Tuning of Laser Emissions in a Full Visible Spectral Range

**DOI:** 10.3390/ijms12032007

**Published:** 2011-03-21

**Authors:** Mi-Yun Jeong, Jeong Weon Wu

**Affiliations:** 1 Department of Physics and Research Institute of Natural Science, Gyeongsang National University, Jinju 660-701, Korea; 2 Department of Physics and Quantum Metamaterials Research Center, Ewha Womans University, Seoul 120-750, Korea

**Keywords:** tunable laser, photonic crystal, wedge cell, cholesteric liquid crystal laser

## Abstract

In order to achieve a continuous tuning of laser emission, the authors designed and fabricated three types of cholesteric liquid crystal cells with pitch gradient, a wedge cell with positive slope, a wedge cell with negative slope, and a parallel cell. The length of the cholesteric liquid crystal pitch could be elongated up to 10 nm, allowing the lasing behavior of continuous or discontinuous spatial tuning determined by the boundary conditions of the cholesteric liquid crystal cell. In the wedge cell with positive slope, the authors demonstrated a continuous spatial laser tuning in the near full visible spectral range, with a tuning resolution less than 1 nm by pumping with only a single 355 nm laser beam. This continuous tuning behavior is due to the fact that the concentration of pitch gradient matches the fixed helical pitch determined by the cell thickness. This characteristic continuous spatial laser tuning could be confirmed again by pumping with a 532 nm laser beam, over 90 nm in the visible spectral range. The scheme of the spatial laser tuning in the wedge cell bearing a pitch gradient enabled a route to designing small-sized optical devices that allow for a wide tunability of single-mode laser emissions.

## 1. Introduction

Since I. P. Il’chishin *et al.* [1] first reported the lasing operation in dye-doped cholesteric liquid crystals (CLC), as a one-dimensional photonic crystal laser system, their subsequent and increased study has yielded much useful data [2–6]. The photonic band gap (PBG) property of CLC could be applied to the tunable band pass filter, lasing, and reflective color display devices, to name but a few. Cholesteric liquid crystals consist of highly birefringent nematic liquid crystals and chiral molecules, exhibiting a self-organized periodic helical structure, which results in a periodic modulation on the refractive index. For the circularly polarized light possessing the same handedness as that of the CLC, a Bragg reflection occurs at the central wavelength of λ_B_ = n × p with a bandwidth of *Δλ* = *p* × *Δn*, where *p* is the helical pitch, $n=\sqrt{(n_{e}^{2}+b_{o}^{2})/2}$ the average refractive index, and *Δn* = *n_e_* − *n_o_* the birefringence of the nematic molecules [7]. The spectral tunability with a high wavelength resolution in the broad spectral range is very useful and necessary for application of single-mode CLC lasers towards absorption spectroscopy, fluorescent excitation, and telecommunications.

In order to realize tunable low-threshold CLC laser devices, many strategies have been applied: changing either pitch or refractive index of the CLC; photo-isomerization method of an optically active chiral agent on a dye-doped CLC (DDCLC) or polymerizing CLC by UV light exposure [8–10]; pitch and refractive index change by temperature change [11–15]; applying a mechanical stress on a CLC elastomer [14]; applying an electric field on ferroelectric liquid crystals [16]; building a spatial pitch gradient in the DDCLC cell [17]. Also it has been reported that a control of dye dopant concentration and an introduction of defect layers in CLC layer, permit the laser wavelength tuning [18–20].

In all the above examples, however, because the CLC cells are composed of two parallel alignment layers with a thin film thickness *d* of several to ∼20 μm, the generated single-mode laser lines take on discrete spectral values, separated by the free spectral range (*Δλ*)*_fsr_* ≈ *λ^2^/(2 × n_f_* *× d)*. Until now, a spectral tuning resolution of *Δλ* *≈ 8 nm* has been achieved [17].

Recently, the authors demonstrated a continuous spatial laser tuning, with a tuning resolution less than 1 nm in the broad visible spectral range, by implementing a gradual change in the optical helical pitch in the wedge cell structure [21]. In this paper, the authors report a continuous spatial laser tuning with a tuning resolution less than 1 nm in the near full visible spectral range, from 474.70 nm to 676.12 nm, by pumping with only a single 355 nm laser beam. By adjusting the dye concentration and combination of the CLCs, the authors could increase the tuning range of laser spectra. Repeating of the lasing experiment on other wedge cells with different CLCs by pumping with a 532 nm laser beam yielded the same lasing behavior, with a continuous spatial tuning in the spectral range over 90nm in the visible spectra. Further study of the continuous laser tuning mechanism in the wedge cell with three types of cholesteric liquid crystal cells revealed that the length of the pitch length could be elongated up to 10 nm, and the behavior of the continuous or discontinuous laser tuning could be determined by the boundary conditions within the CLC cells.

## 2. Experimental Section Sample Preparation

**Figure 1.**
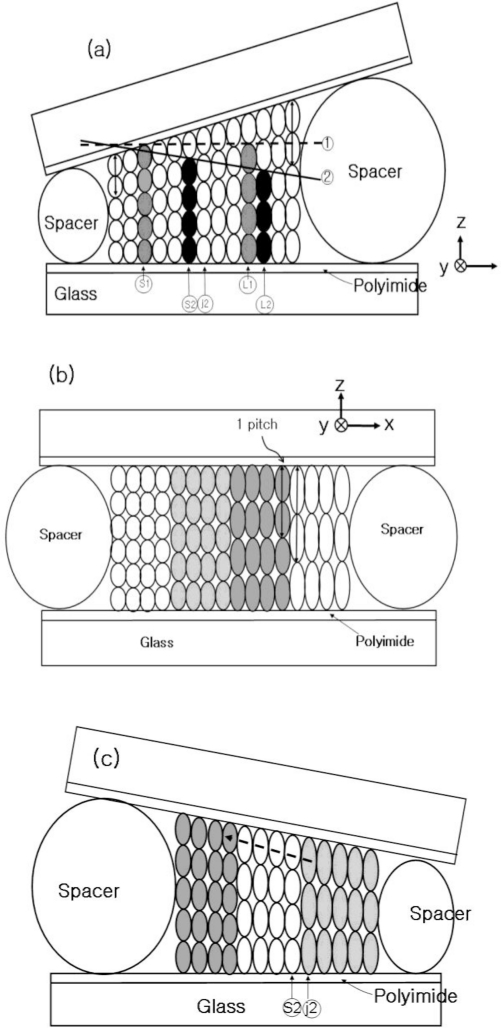
Schematic diagram of the CLC pitch gradient developed in a wedge cell with positive slope (**a**), parallel cell (**b**), and wedge cell with negative slope (**c**).

The authors fabricated three types of cholesteric liquid crystal cells with a pitch gradient, a wedge cell with positive slope (Figure 1a), a wedge cell with negative slope (Figure 1c), and a parallel cell (Figure 1b). In order to fabricate the wedge cells, two different sizes of spacers (thin, 8.25 μm; thick, 12.0 or 15.0 μm) were employed, and a spatial gradient with a thickness change from 1.5 to 2.0 μm over a lateral distance of 1.0 cm is achieved (Figure 1). The concentration gradient of the wedge cell with a positive (negative) slope was established by half-filling with a high (low) chiral dopant concentration CLC doped with two or three laser dyes at the thin (thick) spacer position, respectively. As a control, a parallel CLC cell (P-cell) with a uniform thickness, prepared with a 12.0 μm spacer, was filled in the same manner as the CLC wedge cell. The CLC cells were then kept at room temperature for one to four weeks to develop a pitch gradient through diffusion of the helical rotatory power. Diffusion process to form a stable pitch gradient depends on several parameters, such as chiral dopant concentration, conformational shape of laser dye molecule, viscosity of CLC molecule, and ambient temperature. In case of Coumarin540A and Rhodamin590, we found that four weeks of elapse time is required to have a homogeneous distribution of laser dye in the helical structure of CLC. As an alignment layer, SE-5291 polyimide (pretilt angle of 6°∼7°, Nissan Chemical Korea Co. Ltd., Korea) was employed to fabricate the CLC cells.

In order to cover the full visible spectral range, five different laser dyes were employed. According to dye combinations, three kinds of CLC wedge cells, namely, WL-cell, WM-cell, and WS-cell, were fabricated. WL-cell (DCM (Aldrich, USA) and LDS698 (Exciton, USA), in ≈2 wt%); WM-cell (Coumarin500 (Exciton), Coumarin 540A (Aldrich), and Rhodamine 590 (Exciton), in ≈1 wt%); WS-cell (Coumarin 500 (Exciton), in ≈1 wt%, Coumarin540A (Aldrich), in ≈0.4 wt%, and Rhodamine 590 (Exciton), in ≈0.2 wt%). For the CLC materials, CLC-x1 (λ_B_ = 450 nm) and CLC-x2 (λ_B_ = 670 nm), nematic liquid crystal ZLI2293 and chiral dopant S811 (all from Merck, Germany) were employed.

As an optical pumping source, third harmonic generation 355 nm light and second harmonic generation 532 nm from a Q-switched Nd:YAG laser (pulse width of 7 ns and repetition rate of 10 Hz) were employed. The pumping laser beam with ∼2 mm beam diameter was focused by a lens with a focal length of 20 cm and a beam waist (*w*) at the focal point is calculated using the formula ω = λ/sinθ = 71 μm (where, λ is the wavelength of the pump beam and sinθ = 1/200). In order to attain a strong absorption of optical pumping [19], the focused pump beam was adjusted to be incident obliquely on the sample with an incidence angle of 10°∼45°. Accordingly, the beam size on the sample could be increased up to ∼1.5 times. It is expected that a smaller pump spot size will provide a narrower linewidth with a higher spectral resolution, while a change in the wedge angle does not affect the characteristics of spatial tuning. The generated laser emission along the normal of the CLC cell was collected by a spectrophotometer with a resolution of 0.36 nm (HR 2000+, Ocean Optics, USA).

Figure 1 shows the schematic diagram of the CLC pitch gradient developed in the CLC cells. The cholesteric helical pitches were quantized with the number of half-turns by the boundary condition [22]. The characteristics of pitch gradient formed in the CLC cell is described in Reference [21].

In the wedge cell with a positive slope (Figure 1a) along the positive x-direction, a linear increase in the helical pitch continuously occurred, that is, the pitch gradient fits with the positive slope of the cell thickness. However, if the CLC pitch gradient was developed in a parallel cell along the dashed guideline 1 in Figure 1a [21], only two (S1 and L1) discontinuous pitches (gray color) were allowed by the boundary condition. The pitches between S1 and L1 were elongated to fit their length to the long L1 pitch, Figure 1b. There were discontinuous pitch jumps. The energy was minimized when the pitch was elongated between S1 and L1. Similarly, if the pitch gradient was developed with a negative slope along the solid line 2, Figure 1a by half-filling with a high (low) chiral dopant concentration CLC at a thick (thin) spacer position, discontinuous pitches (S2 and L2, black color) were allowed by the boundary condition. The pitches between the S2 and L2 were subsequently elongated to satisfy the geometrical thickness of the wedge cell thickness, Figure 1c. There was a discontinuous pitch jumping between S2 and J2 (Figure 1c) that lead to the behavior of reverse laser tuning (Figure 7c).

## 3. Results and Discussion

**Figure 2.**
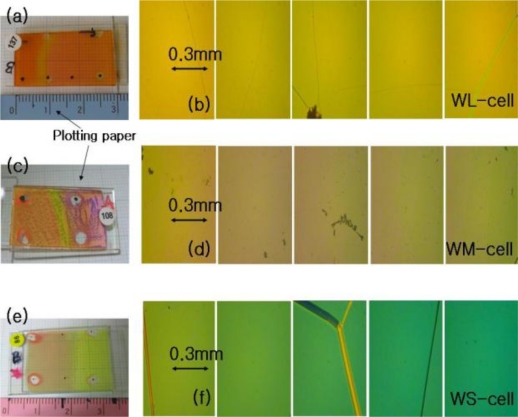
Photographs and juxtapositions of five pieces of polarized microscope images at different spatial positions of the WL-cell (**a** and **b**), the WM-cell (**c** and **d**), and WS-cell (**e** and **f**), respectively.

**Figure 3.**
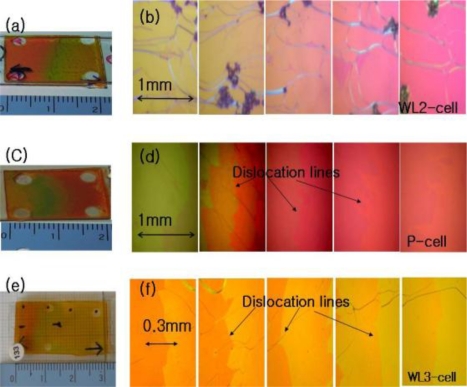
Photographs and juxtapositions of five pieces of polarized microscope images at different spatial positions of the WL2-cell (**a** and **b**), P-cell (**c** and **d**), and WL3-cell (**e** and **f**) respectively.

Figures 2 and 3 show the photographic images and juxtapositions of five pieces of polarized microscope images at different spatial positions within the CLC-cells, respectively. When the photographic images are compared, the wedge cells with positive slope (WL-, WM-, WS-, and WL2-cell), parallel cell (P-cell), and the wedge cell with negative slope (WL3-cell) appear to be similar, with the exception of the WM-cell and WL2-cell, where some signature of dye aggregation due to a high dye concentration should be noted, Figures 2c,d and Figure 3a. When examined with a polarization microscope, in Figure 2b,d,f and Figure 3b, the wedge cells with positive slope show a continuous color change, stemming from a continuous pitch change. However, the parallel cell (P-cell) and wedge cell with negative slope (WL3-cell), in Figure 3d,f, show discontinuous color change when crossing Cano lines (or dislocation lines) in each of the polarized microscope images. Cano lines originate from a mismatch between the CLC pitch and the helical pitch determined by the cell thickness. This discontinuous color change is related to the discontinuous pitch change of the CLC (Figure 1b,c); discontinuous pitch jumping leads to discontinuous laser tuning (Figure 7b,c). However, when the concentration of pitch gradient fits with the helical pitch determined by the cell thickness with positive slope, Cano lines disappear, which is similar to the wedge cell with positive slope, (Figure 2b,d,f).

**Figure 4.**
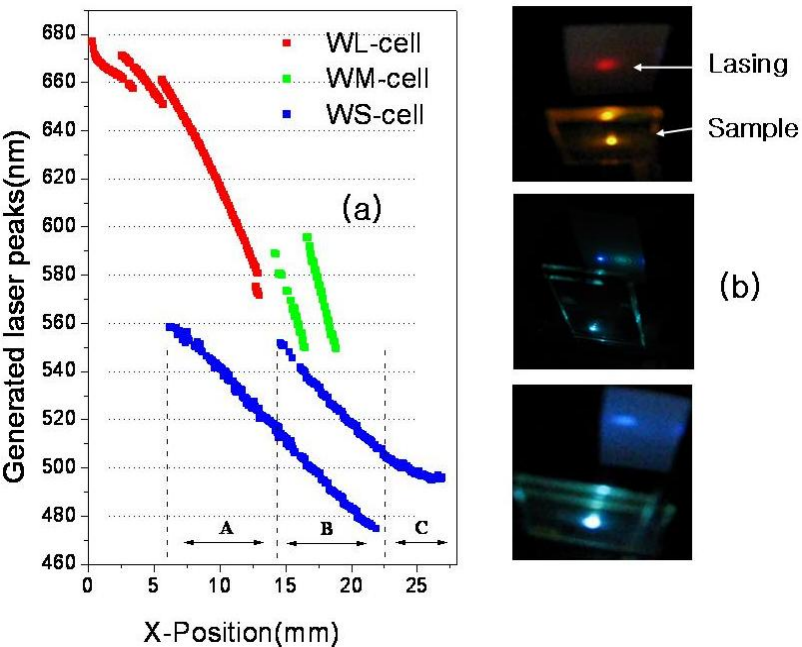
(**a**) Laser lines of the wedge cells as a function of spatial position for each WL-, WM-, and WS-cell, respectively; (**b**) Photographs of red, green and blue lasing.

Figure 4 shows the laser lines of the wedge cells with positive slope as a function of spatial position with the inset of the lasing photographs for each WL-, WM-, and WS-cell, respectively. In the WL-cell (DCM and LDS698 dyes were added), a continuous tuning of the laser was achieved over a range of 97 nm, from 580.69 nm to 676.91 nm, with an accuracy of Δλ ≈ 1 nm, by a 100 μm spatial movement of the cell in the x-direction (Figure 4a). This continuous tuning behavior is due to the fact that the concentration of pitch gradient fit with the helical pitch determined by the cell thickness (see Figure 1a).

In fabricating WM-cell, three different laser dyes were dissolved in CLCs with both high and low chiral dopant concentrations. The decrease in the spectral range of laser tunability is due to the fluorescence quenching coming from laser dye aggregations. When a thick cell was fabricated by employing 32 and 35 μm spacers, the spectral tuning range was 47.6 nm, from 549.8 nm to 595.5 nm. As the x-position of pump beam spot is increased, lasing operation takes place at the short wavelength band edge, and it jumped to the long wavelength band edge. The spectral range of laser tuning for both short and long wavelength band edge was from 550 nm∼596 nm (Figure 4, WM-cell). In WM-cell, we observed that the spatial tuning of laser wavelength followed a discrete step of Δλ ≈ 2.6 nm upon moving the pump beam spot over the distance of 50 μm, which is due to the mismatch between the wedge cell’s thickness gradient and the chiral dopant concentration.

In order to further extend the tuning spectral range to the blue wavelengths, the WS-cell was introduced. In the case of the WS-cell, in order to minimize dye aggregation and maximize the lasing range, the concentration and combination of the adding dyes were adjusted, yielding a high chiral dopant concentration CLC doped with two (Coumarin500 (1.0 wt%), Coumarin540A (0.4%)) laser dyes and a low chiral dopant concentration CLC doped with three (Coumarin500 (1.0 wt%), Coumarin540A (0.4 wt%) and Rhodamine590 (0.2 wt%)) laser dyes. Then, a broad continuous laser tuning range was achieved with the spectral width exceeding 84.02 nm, from 474.70 nm to 558.02 nm. The generated laser wavelength can be spatially tuned with the resolution of Δλ ≈ 1 nm, by a 200 μm movement in the x-direction. In this lasing action, the Forster energy transfer [23] from Coumarine500 (sensitizer) to Coumarine540A and Rhodamine590 is involved.

**Figure 5.**
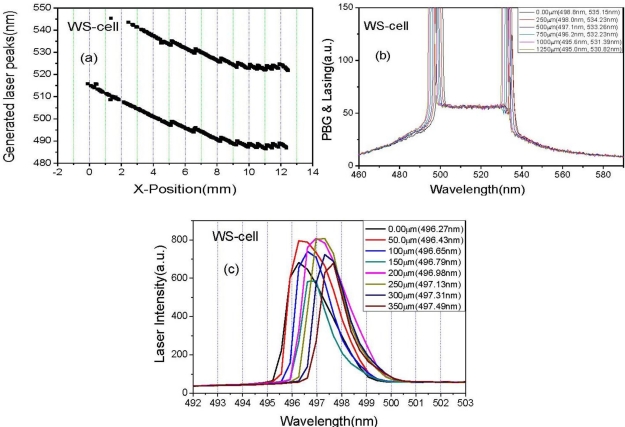
(**a**) Laser lines of the WS-cell as a function of spatial position; (**b**) Reflection spectra and lasing spectrum change obtained by the scanning distance of 250 μm; (**c**) lasing spectrum change obtained by the scanning distance of 50 μm.

In order to attain the spectral resolution of laser tuning as high as Δλ ≤ 0.3 nm, the scanning distance of pump beam spot should be decreased, which can be seen in Figure 5, where Δλ ≈ 1 nm is attained for scanning distance of 250 μm, Figure 5b, and Δλ ≈ 0.2 nm is attained for the scanning distance of 50 μm (Figure 5c).

**Figure 6.**
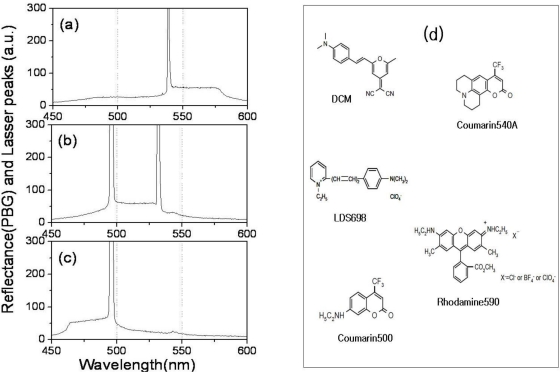
Spectra of the reflectance (PBG) and laser peaks of the WS-cell for area A (**a**); area B (**b**); and area C (**c**) in Figure 4 (**a**); respectively. Molecular structures of the laser dyes (**d**).

In order to study the relationship between lasing position and photonic band gap (PBG), we simultaneously measured PBG and lasing of the samples with a spectrophotometer (OOI, Ocean optics instrument: HR 2000+, USA). Near the PBG edges, since the group velocity *v*_g_, becomes near to zero and the number of the density of mode (DOM) increases remarkably, a lasing operation occurs. In the case of the WL-cell, since the linearly shaped rod-like DCM and LDS698 molecules (Figure 6d) prefer to align parallel to the nematic director, the CLC laser cavity in the longer wavelength band edge of the PBG was much more efficient in amplifying the stimulated emission of fluorescence, so the lasing operation occurred at the longer wavelength band edge of the PBG, Figure 5a. However, in the cases of the WM- and WS-cells, when the T-shaped Rhodamine590 and the plane-shaped Coumarin540A molecules (Figure 6d) aligned parallel to the nematic director, both the longer and the shorter wavelength band edges of the PBG could be efficient in amplifying the stimulated emission of fluorescence, so the lasing operation could occur at both sides of the PBG edges. Figure 6a–c shows the spectra of the PBG and lasing of areas A, B, and C of the WS-cell of Figure 4, respectively. In area A (Figure 6a), lasing is generated at the shorter wavelength PBG edge. In area C (Figure 6c), the lasing is generated at the longer wavelength PBG edge. In area B (Figure 6b), lasing is generated at both longer and shorter wavelength PBG edges. The missing lasing at the longer (or shorter) wavelength PBG edge is due to the fact that the lasing spectral range is limited by the fluorescence range of the dyes, Figure 6a (in Figure 6c).

**Figure 7.**
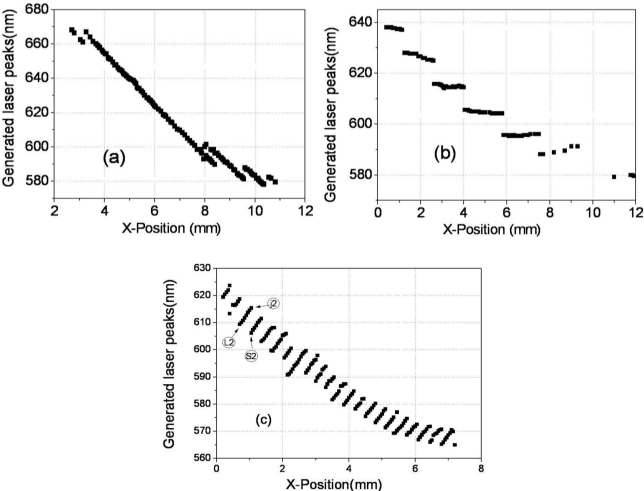
Laser lines as a function of spatial position for the wedge cell with a positive slope, WL2-cell (**a** and Figure 1a), for the parallel cell (**b** and Figure 1b), and for the wedge cell with a negative slope (**c** and Figure 1c).

**Figure 8.**
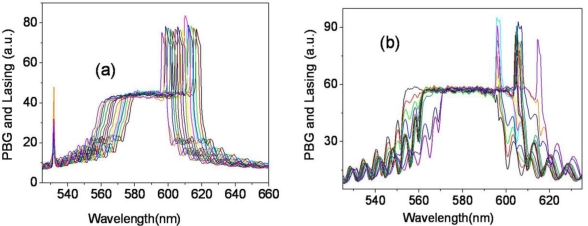
PBG and lasing spectrum change by x-position movement of the WL2-cell (**a**) and P-cell (**b**).

The lasing could also be generated by a 532 nm laser pump and the same lasing behavior is observed in another wedge cell, WL2-cell (DCM and LDS698 dyes were added). Similar to the results of the WL-cell pumped by 355 nm, a continuous tuning of the laser wavelength is achieved in a range over 90 nm, from 578.22 nm to 668.14 nm in the WL2-cell, (Figure 7a). The laser wavelength is tuned continuously without any structural deformation of band edges of the reflection spectrum, Figure 8a. In order to study the mechanism of the continuous laser tuning in the wedge cell with a pitch gradient, we compared the lasing results of the three types of CLC cells, the wedge cell with positive slope (Figure 7a), the parallel cell (Figure 7b), and the wedge cell with negative slope (Figure 7c). There is a drastic difference in the tuning characteristics among them. In the P-cell, eight discrete laser spectral lines were observed, which were separated by a laser tuning difference of Δλ ≈ 10 nm in a 12 μm thick cell. Even in the presence of a continuous concentration gradient of chiral dopants, a discrete tuning of the laser lines appears at the spatial CLC cell positions to satisfy the boundary condition. Between the two laser lines (Figure 8b), both the long and short edges of the stop band reflection spectrum suffer strong structural deformations, due to the fact that the pitch of the concentration gradient does not match with the fixed helical pitch determined by the cell thickness. In the case of the wedge cell with a negative slope, where the concentration gradient was developed with a negative slope in the wedge cell (Figure 1c), the laser spectra were tuned discontinuously in the shape of a toothed wheel due to the boundary conditions.

The incident pumping energy was 5∼10 μJ/pulse at 355 nm and 20∼40 μJ/pulse at 532 nm, respectively. Optical conversion efficiency of the lasing was about 1∼2%. If we combine external cavity method [24–25] into our wedge cell structure, we can improve the conversion efficiency and reduce divergence angle of the laser beam.

## 4. Conclusions

In order to study the mechanism of the continuous laser tuning in CLC cells, the authors designed and fabricated three types of cholesteric liquid crystal cells, a wedge cell with a positive slope, a wedge cell with a negative slope, and a parallel cell, and found that the length of the pitch length could be elongated up to 10 nm, and the behavior of continuous or discontinuous laser tuning is determined by the boundary conditions of the CLC cells. From the wedge structured cholesteric liquid crystal films with a spatial pitch gradient, the authors demonstrated a continuous spatial laser tuning in the full visible spectral range, with a tuning resolution of Δλ ≤ 1 nm by pumping with only a single 355 nm laser beam. The continuous tuning is made possible by the fact that the concentration of pitch gradient fits with the helical pitch determined by the cell thickness with positive slope. The continuous spatial laser tuning behavior could be confirmed again by pumping with a 532 nm laser beam, over 90 nm in the visible spectral range. Furthermore, the PBG also could be shifted spatially with a similar tuning resolution. This scheme of the spatial laser tuning in a wedge cell with a pitch gradient opens the way to designing small-sized optical devices that allow for a wide tunability with high tuning resolution of single-mode laser emissions.

## References

[1] Il’chishin IP, Tikhonov EA, Tishchenko VG, Shpak MT (1980). Generation of a tunable radiation by impurity cholesteric liquid crystals. JETP Lett.

[2] Kopp VI, Zhang Z-Q, Genack AZ (2003). Lasing in chiral photonic structures. Prog. Quantum Electron.

[3] Schmidtke J, Stille W (2003). Fluorescence of a dye-doped cholesteric liquid crystal film in the region of the stop band: theory and experiment. Eur. Phys. J. B.

[4] Ozaki M, Matsuhisa Y, Yoshida H, Ozaki R, Fujii A (2007). Photonic crystals based on chiral liquid crystal. Phys. Stat. Sol.

[5] Ford AD, Morris SM, Coles HJ (2006). Coles Photonics and lasing in liquid crystals. Mater. Today.

[6] Finkelmann H, Kim ST, Munoz A, Palffy-Muhoray P, Taheri B (2001). Tunable mirrorless lasing in cholesteric liquid crystalline elastomers. Adv. Mater.

[7] Yeh P, Gu C, Joseph WG (1999). Optical properties of cholesteric LCs. Optics of Liquid Crystal Displays.

[8] Chanishivili A, Chilaya G, Petriashvili G, Barberi R, Bartolino R, Cipparrone G, Mazzulla A, Orieol L (2004). Lasing in dye-doped cholesteric liquid crystals: Two new tuning strategies. Adv. Mater.

[9] Huang Y, Chen L-P, Doyle C, Zhou Y, Wu S-T (2006). Spatially tunable laser emission in dye-doped cholesteric polymer films. Appl. Phys. Lett.

[10] Bobrovsky AY, Boico NI, Shibaev VP, Wendorff JH (2003). Photo-patternable cholesteric materials. Avd. Mater.

[11] Funamoto K, Ozaki M, Yoshino K (2003). Discontinuous shift of lasing wavelength with temperature in cholesteric liquid crystal. Jpn. J. Appl. Phys.

[12] Huang Y, Zhou Y, Wu S-T (2006). Spatially tunable laser emission in dye-doped photonic liquid crystals. Appl Phys Lett.

[13] Huang Y, Zhou Y, Doyle C, Wu S-T (2006). Tuning photonic band gap in cholesteric liquid crystals by temperature-dependent dopant solubility. Opt. Express.

[14] Finkelmann H, Kim ST, Muaoz A, Palffy-Muhoray P, Taheri B (2001). tunable mirrorless lasing in cholesteric liquid crystalline elastomers. Adv. Mater.

[15] Ozaki M, Kasano M, Ganke D, Haase W, Yoshino K (2002). Mirrorless lasing in a dye-doped ferroelectric liquid crystal. Adv. Mater.

[16] Yu H, Tang BY, Li J, Li L (2005). Electrically tunable lasers made from electro-optically active photonics band gap materials. Opt. Express.

[17] Manabe T, Sonoyama K, Takanishi Y, Ishikawa K, Takezoe H (2008). Toward practical application of cholestric liquid crystals to tunable lasers. J. Mater. Chem.

[18] Belyakov VA (2006). DFB Lasing in Chiral LC at diffraction of pumping wave. Ferroelectrics.

[19] Belyakov VA (2006). Low threshold DFB lasing in chiral LC at diffraction of pumping wave. Mol. Cryst. Liq. Cryst.

[20] Gevorgyan AH, Oganesyan KB, Harutyunyan EH, Arutyunyan SO (2010). The peculiarities of radiation of chiral photonic crystals with isotropic defect layer. Opt. Commun.

[21] Jeong M-Y, Wu JW (2010). Continuous spatial tuning of laser emissions with tuning resolution less than 1 nm in a wedge cell of dye-doped cholesteric liquid crystals. Opt. Express.

[22] De Gennes PG, Prost CJ (1993). The Physics of Liquid Crystals.

[23] Chambers M, Fox M, Grell M, Hill J (2002). Lasing from a Forster transfer fluorescent dye couple dissolved in a chiral nematic liquid crystal. Adv. Funct. Mater.

[24] Zhou Y, Huang Y, Wu S-T (2006). Enhancing cholesteric liquid crystal laser performance using a cholesteric reflector. Opt. Express.

[25] Zhou Y, Huang Y, Ge Z, Chen LP, Hong Q, Wu TX, Wu S-T (2006). Enhanced photonic band edge laser emission in a cholesteric liquid crystal resonator. Phys Rev E.

